# The role of composite dietary antioxidants in elderly cognitive function: insights from NHANES

**DOI:** 10.3389/fnut.2024.1455975

**Published:** 2024-09-25

**Authors:** Fangsen Chen, Junhan Chen, Peitian Liu, Yanling Huang

**Affiliations:** ^1^Department of Endocrinology and Metabolism, Zhongshan Hospital of Xiamen University, School of Medicine, Xiamen University, Xiamen, China; ^2^The School of Clinical Medicine, Fujian Medical University, Fuzhou, China

**Keywords:** composite dietary antioxidant index, cognitive function, older adults, antioxidant intake, NHANES

## Abstract

**Objective:**

This study investigates the relationship between the Composite Dietary Antioxidant Index (CDAI) and cognitive function among elderly individuals, aiming to understand how increased antioxidant intake affects cognitive abilities in an aging population.

**Methods:**

Utilizing data from the National Health and Nutrition Examination Survey (NHANES) from 2011 to 2014, we analyzed a sample of 2,516 participants aged 60 and above. Cognitive performance was assessed using the CERAD Word Learning and Recall Test, the Animal Fluency Test, and the Digit Symbol Substitution Test. Multivariable regression models were adjusted for demographic, dietary, and health-related factors to explore the association between CDAI scores and cognitive outcomes.

**Results:**

The regression analyses showed a statistically significant positive association between higher CDAI scores and cognitive performance across several tests. Specifically, increments in CDAI were associated with increased scores in the CERAD Word Learning Test: Score 1 (*β* = 0.04, 95% CI [0.03, 0.06]), Score 2 (*β* = 0.04, 95% CI [0.02, 0.05]), Score 3 (*β* = 0.04, 95% CI [0.02, 0.06]), and the Delayed Recall Test (*β* = 0.04, 95% CI [0.01, 0.06]). Additionally, significant improvements were observed in the Animal Fluency Test (*β* = 0.19, 95% CI [0.14, 0.24]) and the Digit Symbol Test (*β* = 0.55, 95% CI [0.39, 0.71]). Subgroup analyses further highlighted that higher CDAI scores conferred more pronounced cognitive benefits in women, individuals aged 80 and above, Non-Hispanic Black people, and those with lower educational levels, suggesting that dietary antioxidants might be particularly beneficial in these groups.

**Conclusion:**

An antioxidant-rich diet may represent a viable intervention to mitigate age-related cognitive decline, supporting cognitive health in the elderly. These results underscore the potential public health implications of dietary recommendations aimed at increasing antioxidant consumption among older adults. Further studies are necessary to confirm these findings and to investigate the underlying mechanisms in detail.

## 1 Introduction

The global aging population has intensified the focus on cognitive health as a significant public health issue. Dementia is often characterized by cognitive decline. WHO estimates 46.8 million dementia cases globally in 2015, expected to surge to 131.5 million by 2050 (1). The causes of cognitive decline are complex, involving genetic, environmental, physiological, psychological, social, lifestyle, and dietary factors (2–5). The significance of identifying changeable risk factors associated with cognitive function is growing. Numerous research looks at the connection between cognitive performance and food. These factors may help slow cognitive decline during aging and prevent or delay cognitive impairment or dementia (6–8).

Oxidative and inflammatory damage are crucial aspects of the multifaceted pathophysiological mechanisms of cognitive decline (9–11). As a result, inadequate consumption of antioxidants in the diet might be a changeable risk factor for cognitive deterioration. Earlier research has indicated that antioxidants in the diet can inhibit the generation of oxygen-rich compounds and potentially decrease oxidative DNA damage (12). As free radicals increase with age, antioxidants can mitigate the destructive impact of free radicals on neurons, thereby delaying cognitive decline (13).

An accurate and dependable nutritional technique for evaluating the diet’s total antioxidant content is the CDAI. It consists of the following six dietary antioxidants: carotenoids, selenium, zinc, vitamins A, C, and E (14–16). Prior research has connected CDAI to depression (17) and colorectal cancer (16). Although Prior studies have generally focused on the link between specific antioxidants and cognitive outcomes, there has been limited investigation into the potential combined benefits of antioxidants on cognitive well-being (18). Thus, this research aims to investigate the cross-sectional relationship between the Composite Dietary Antioxidant Index (CDAI) and cognitive function in older adults, utilizing data from the 2011–2014 NHANES.

## 2 Experimental materials and process

### 2.1 Study methodology and individuals

This study analyzed data specifically from the 2011–2014 cycles of the National Health and Nutrition Examination Survey (NHANES), a biennial survey conducted by the National Center for Health Statistics (NCHS) since 1999. NHANES evaluates the physical well-being and dietary condition of individuals in the United States.

**Figure 1:**
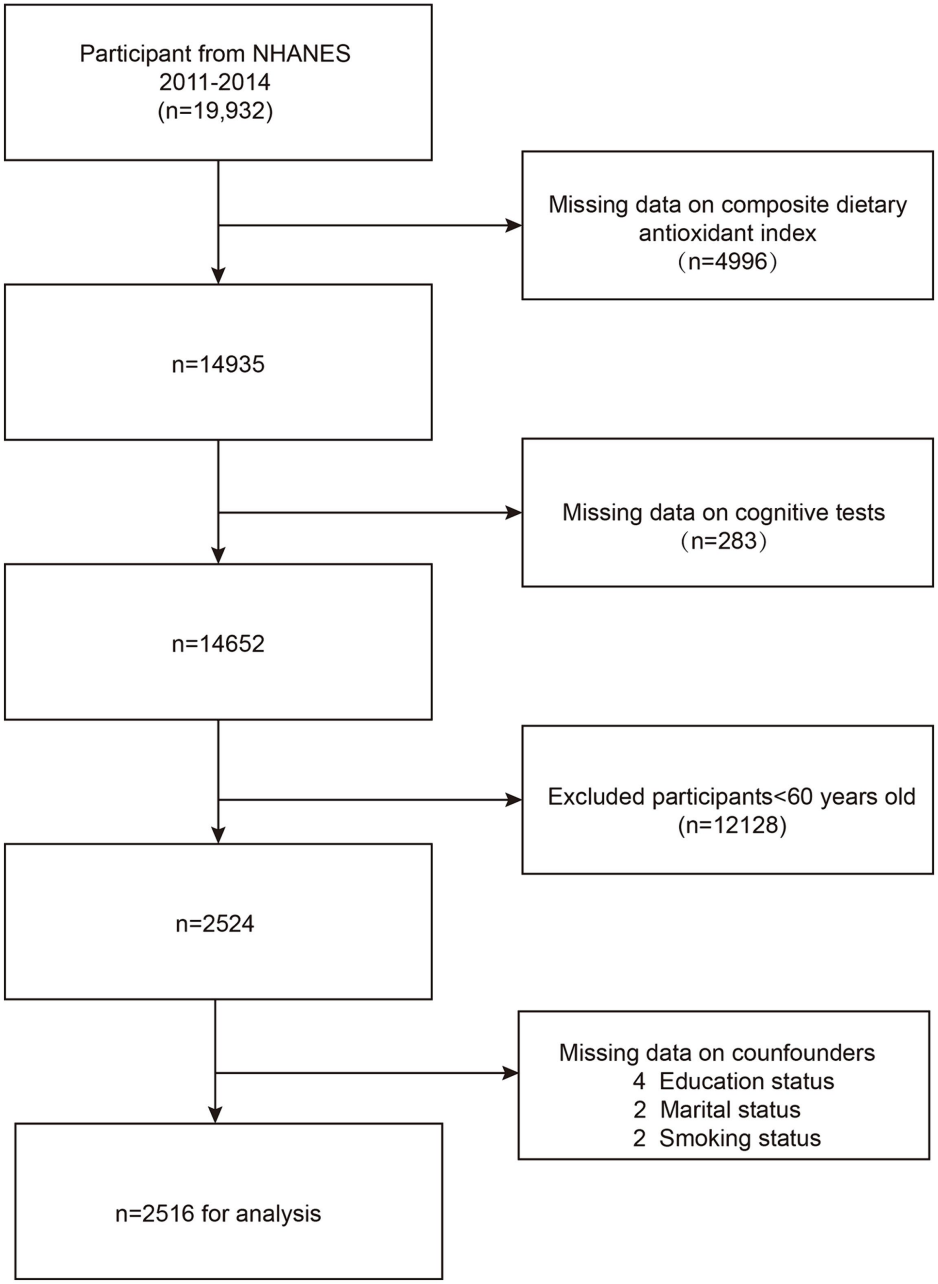
Flow chart of participants selection. NHANES, National Health and Nutrition Examination Survey.

We excluded 4,996 participants due to incomplete CDAI data and 283 participants with missing cognitive outcomes. Additionally, 12,128 participants under the age of 60 were also excluded. Eight participants with missing covariates, such as education level, marital status, and habit of smoking were further eliminated. The ultimate research group comprised 2,516 people. The sample selection process and results are detailed in Figure 1.

### 2.2 Definition of composite dietary antioxidant index

The NHANES survey gathered nutritional data by conducting two 24-h recall surveys. The first had been a face-to-face interview in the mobile Examination Center, and the following interview was conducted 3 to 10 days subsequently via phone, involving recalling food and beverage intake from the previous 24 h (19).

The CDAI is a nutritional technique used to analyze the overall antioxidant qualities of a diet. It is computed using the dietary intake of six antioxidants: zinc, selenium, carotenoids, vitamins A, C, and E (14, 15). In this research, the carotenoids were collected by determining the mean consumption of alpha-carotene, beta-carotene, beta-cryptoxanthin, lycopene, lutein, and zeaxanthin across the two recall periods (20).

In short, we achieved standardization of the six dietary antioxidants by calculating the difference between the intake of each antioxidant and its average, and then dividing by the standard deviation (16).

The specific formula is as follows:


$$\mathrm{CDAI}=\overset{6}{\underset{i=1}{\sum }}\frac{x_{i}-u_{i}}{s_{i}}$$

$x_{i}$ represents the daily antioxidant intake, $u_{i}$ represents the average value of $x_{i},s_{i}$ is the standard deviation of $u_{i}$ .

### 2.3 Cognitive outcomes

The NHANES study utilized three distinct tests to measure cognitive function: the CERAD Word Learning and Recall Test, the Animal Fluency Test (AFT), and the Digit Symbol Substitution Test (DSST). The CERAD Word Learning Test assesses the ability to remember new verbal information immediately and after a delay. It consists of three consecutive learning trials and one delayed recall trial. Each trial is evaluated on a scale from 0 to 10. The AFT assesses categorical fluency in language, which is an important part of executive function. In addition, the DSST, which is part of the Wechsler Adult Intelligence Scale (WAIS-III), evaluates the efficiency of processing, continuous focus, and working recall.

### 2.4 Covariates

To evaluate the impact of potential confounders, several key covariates were selected, including sex, age, race, education level, marital status, smoking status, BMI, the poverty-to-income ratio (PIR), physical activity levels, and medication use. These variables were collected through standardized questionnaires, and each participant’s weight and height were obtained through physical examinations. Physical activity levels were calculated by adding time spent per week doing vigorous or moderate work and recreational activities. Additionally, certain other dietary factors, such as choline, docosahexaenoic acid (DHA), and eicosapentaenoic acid (EPA) intake, were evaluated as potential covariates. The NHANES website^1^ provides detailed explanations of how these variables were calculated.

### 2.5 Statistical analysis

Statistical analyses adhered to Centers for Disease Control and Prevention guidelines, employing NHANES sample weights to take into account the survey’s complexity. The continuous data were reported using the mean ± SE, while the categories were shown as proportions. The participants were categorized into quartiles based on their CDAI scores. Weighted linear regression was used to analyze differences between groups for continuous variables, while chi-square tests were employed for categorical variables.

Three multivariable regression models were used to investigate the correlation between CDAI and cognitive scores. Model 1: Not modified; Model 2: Modified to account for sex, age, and race; Model 3: Additionally controlled for education, marital status, PIR (personal income ratio), BMI (body mass index), smoking, cholesterol, glycohemoglobin, physical activity levels, and medication use.

The subgroup analyses were categorized based on variables including sex (male/female), age (60–70 years, 70–80 years, ≥80 years), race/ethnicity (Mexican American, Non-Hispanic Black people, Non-Hispanic White people, other), education level (less than high school, high school or higher), poverty-to-income ratio (PIR; ≤1, 1–2, 2–4, ≥4), and BMI (≤25, 25–30, ≥30) to study the link between CDAI and cognitive function. Smoothing curve fitting was employed to address nonlinear relationships.

All analyses were conducted using EmpowerStats (2.0)^2^ and R software,^3^ utilizing MEC weights. *p*-values<0.05 were considered statistically significant.

## 3 Results

### 3.1 Baseline characteristics

The basic features of the participants are presented in Table 1. Compared to the Q1 group, participants with higher CDAI scores were more likely to be male, Non-Hispanic White, married, and to have higher educational levels. Participants in the Q4 group also had higher income levels, vitamin and mineral intakes. Additionally, they had higher cognitive test scores. In the highest quartile, participants had significantly higher antioxidant intake, including vitamin A (1131.46 ± 1287.61 μg/day), vitamin C (135.60 ± 96.00 mg/day), vitamin E (12.78 ± 5.71 mg/day), zinc (13.87 ± 4.79 mg/day), selenium (131.95 ± 43.71 μg/day), and total carotenoids (17516.18 ± 18467.07 μg/day), along with lower glycohemoglobin levels. There were no notable disparities identified among the groups in terms of age, smoking status, BMI, liver enzymes, and cholesterol levels.

**Table 1:** Characteristics of the study population according to CDAI quartiles.

Variable	Q1	Q2	Q3	Q4	*p* value
	(−7.9–−2.5), *N* = 629	(−2.5–−0.6), *N* = 629	(−0.6–1.8), *N* = 629	(1.8–69.7), *N* = 629	
Age (years)	69.44 ± 6.97	69.56 ± 6.73	69.34 ± 6.63	68.79 ± 6.64	0.1814
Gender (%)					<0.0001
Male	29.97	38.92	51.37	54.60	
Female	70.03	61.08	48.63	45.40	
Race and ethnicity (%)					0.0042
Mexican American	4.05	3.18	2.87	3.06	
Non-Hispanic White people	73.31	80.74	83.01	81.70	
Non-Hispanic Black people	12.93	7.87	6.66	7.36	
Other	9.71	8.20	7.45	7.89	
Educational attainment (%)					<0.0001
Less than high school	23.76	18.81	14.46	12.94	
High school diploma	24.70	25.79	19.51	17.64	
More than high school	51.54	55.39	66.03	69.42	
Marital status (%)					<0.0001
Married	58.97	59.38	69.46	70.53	
Single or separated	41.03	40.62	30.54	29.47	
Smoking status (%)					0.3479
Yes	51.82	53.13	48.66	49.50	
No	48.18	46.87	51.34	50.50	
Family poverty income ratio	2.70 ± 1.52	2.85 ± 1.48	3.26 ± 1.54	3.34 ± 1.54	<0.0001
Anti-hypertension therapy (%)	54.36	54.83	52.43	51.78	0.5200
Lipid-lowering therapy (%)	53.35	48.81	51.64	48.21	0.0931
Physical activity levels (%)					0.7977
<60 min	5.21	3.96	3.66	4.59	
160–180 min	11.79	15.0	13.86	13.68	
≥180 min	39.2	36.05	37.08	36.29	
Missing	43.8	44.99	45.4	45.44	
BMI (kg/m^2^)	29.07 ± 6.20	29.72 ± 6.41	29.33 ± 6.39	29.05 ± 6.39	0.2134
Total Energy (kcal/day)	1304.39 ± 311.24	1671.26 ± 332.86	1967.44 ± 401.68	2283.16 ± 529.27	<0.0001
Dietary Vitamin A intake (mg/day)	361.16 ± 162.00	527.38 ± 196.25	679.32 ± 304.55	1131.46 ± 1287.61	<0.0001
Dietary Vitamin C intake (mg/day)	45.08 ± 30.29	71.34 ± 38.86	84.64 ± 44.33	135.60 ± 96.00	<0.0001
Dietary Vitamin E intake (mg/day)	4.62 ± 1.80	6.55 ± 2.16	8.65 ± 2.68	12.78 ± 5.71	<0.0001
Dietary Zinc intake (mg/day)	6.60 ± 1.90	8.69 ± 2.20	10.97 ± 2.93	13.87 ± 4.79	<0.0001
Dietary Selenium intake (mcg/day)	70.11 ± 21.38	91.85 ± 21.49	108.50 ± 27.83	131.95 ± 43.71	<0.0001
Dietary Total carotenoid intake (mcg/day)	3881.15 ± 3468.48	6454.22 ± 4163.23	9279.33 ± 6211.05	17516.18 ± 18467.07	<0.0001
Dietary Alpha-carotene intake (mcg/day)	219.75 ± 388.45	350.17 ± 445.18	446.64 ± 628.21	863.49 ± 2677.87	<0.0001
Dietary Beta-carotene intake (mcg/day)	1028.19 ± 1131.61	1741.06 ± 1602.37	2426.18 ± 2384.11	4992.90 ± 9064.29	<0.0001
Dietary Beta-cryptoxanthin intake (mcg)	51.20 ± 119.11	77.80 ± 159.19	109.57 ± 254.20	149.45 ± 485.17	<0.0001
Dietary Lycopene intake (mcg)	1765.24 ± 2777.87	3077.54 ± 3458.07	4636.30 ± 5363.46	8036.93 ± 9203.02	<0.0001
Dietary Lutein + zeaxanthin intake (mcg)	816.77 ± 989.11	1207.65 ± 1250.23	1660.63 ± 1747.45	3473.41 ± 5975.26	<0.0001
Dietary Total choline intake (mg)	211.60 ± 73.65	283.48 ± 80.74	326.24 ± 94.92	395.95 ± 126.73	<0.0001
Dietary EPA intake (gm)	0.02 ± 0.03	0.03 ± 0.08	0.03 ± 0.07	0.05 ± 0.10	<0.0001
Dietary DHA intake (gm)	0.04 ± 0.12	0.07 ± 0.22	0.08 ± 0.18	0.11 ± 0.27	<0.0001
Total Cholesterol (mmol/L)	5.06 ± 1.17	4.89 ± 1.10	4.90 ± 1.03	4.96 ± 1.11	0.0328
Glycohemoglobin (%)	6.21 ± 1.34	6.13 ± 1.13	6.00 ± 1.01	5.91 ± 0.87	<0.0001
ALT (U/L)	20.99 ± 11.99	21.93 ± 10.69	23.01 ± 14.08	21.94 ± 12.21	0.0409
AST (U/L)	25.24 ± 12.63	25.14 ± 9.55	25.10 ± 9.39	24.42 ± 8.95	0.4601
CERAD: Score Trial 1 Recall	4.49 ± 1.73	4.61 ± 1.66	4.85 ± 1.66	4.92 ± 1.69	<0.0001
CERAD: Score Trial 2 Recall	6.52 ± 1.86	6.67 ± 1.87	6.84 ± 1.82	6.89 ± 1.63	0.0010
CERAD: Score Trial 3 Recall	7.32 ± 1.80	7.40 ± 1.82	7.63 ± 1.81	7.73 ± 1.67	0.0001
CERAD: Score Delayed Recall	5.68 ± 2.38	5.90 ± 2.21	6.13 ± 2.31	6.21 ± 2.05	0.0002
Animal Fluency: Score Total	15.54 ± 5.40	16.51 ± 5.39	17.06 ± 5.16	17.58 ± 5.42	<0.0001
Digit Symbol: Score	42.01 ± 17.47	45.07 ± 17.70	48.20 ± 16.26	50.43 ± 16.03	<0.0001

BMI, body mass index (calculated as weight in kilograms divided by height in meters squared); EPA, Eicosapentaenoic; DHA, Docosahexaenoic; ALT, Alanine aminotransferase; AST, Aspartate aminotransferase. The descriptive statistics are expressed as mean ± standard deviation and percentage for continuous and categorical variables.

### 3.2 Correlation between CDAI and cognitive function

**Figure 2:**
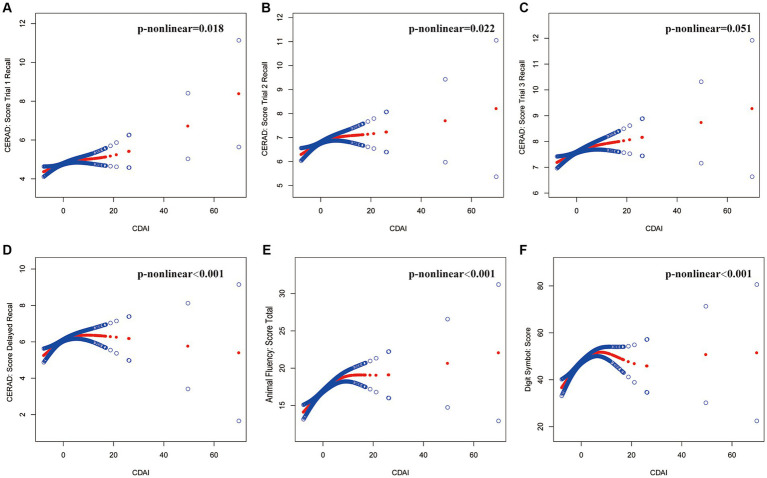
The association between CDAI and cognitive tests. The solid red line represents the smooth curve fit between variables, and the blue bands represent the 95% confidence interval from the fit. **(A)** CERAD: Score Trial 1 Recall, **(B)** CERAD: Score Trial 2 Recall, **(C)** CERAD: Score Trial 3 Recall, **(D)** CERAD: Score Delayed Recall, **(E)** Animal Fluency: Score Total, **(F)** Digit Symbol: Score. Age, gender, race, education, marital status, ratio of family income to poverty, body mass index, smoking status, total cholesterol, and glycohemoglobin were adjusted.

The results of the multivariate regression analysis are presented in Table 2 and Figure 2. In Model 1, the CDAI positively impacts the scores of various cognitive tests. Specifically, the CERAD test shows significant improvements in word learning and recall scores, with *β* values for the first, second, third word tests, and delayed recall being (*β* = 0.04, 95% CI [0.03, 0.06]), (*β* = 0.04, 95% CI [0.02, 0.06]), (*β* = 0.04, 95% CI [0.03, 0.06]), and (*β* = 0.04, 95% CI [0.02, 0.06]), respectively. Both the animal fluency test (*β* = 0.18, 95% CI [0.13, 0.23]) and the digit symbol test (*β* = 0.62, 95% CI [0.46, 0.78]) also show positive associations. In Model 3, this relationship persists, with the *β* values for the cognitive test scores being as follows: first word test (*β* = 0.04, 95% CI [0.03, 0.06]), second word test (*β* = 0.04, 95% CI [0.02, 0.05]), third word test (*β* = 0.04, 95% CI [0.02, 0.06]), delayed recall (*β* = 0.04, 95% CI [0.02, 0.06]), animal fluency test (*β* = 0.19, 95% CI [0.13, 0.24]), and digit symbol test (*β* = 0.55, 95% CI [0.38, 0.71]).

**Table 2:** Association of composite dietary antioxidant index and cognitive tests.

	Model 1	Model 2	Model 3
	β [95% CI] *p*	β [95% CI] *p*	β [95% CI] *p*
**CERAD: Score Trial 1 Recall**			
**CDAI**			
Q1	Ref	Ref	Ref
Q2	0.12 (−0.07, 0.31) 0.22	0.12 (−0.07, 0.32) 0.21	0.12 (−0.08, 0.31) 0.23
Q3	0.36 (0.17, 0.55) <0.01	0.37 (0.17, 0.56) <0.01	0.37 (0.18, 0.56) <0.01
Q4	0.43 (0.24, 0.62) <0.01	0.43 (0.24, 0.63) <0.01	0.44 (0.24, 0.63) <0.01
continuous	0.04 (0.03, 0.06) <0.01	0.04 (0.03, 0.06) <0.01	0.04 (0.03, 0.06) <0.01
P for trend	<0.0001	<0.0001	<0.0001
**CERAD: Score Trial 2 Recall**			
**CDAI**			
Q1	Ref	Ref	Ref
Q2	0.15 (−0.06, 0.35) 0.16	0.14 (−0.06, 0.35) 0.16	0.15 (−0.06, 0.35) 0.16
Q3	0.32 (0.12, 0.53) <0.01	0.32 (0.11, 0.52) <0.01	0.29 (0.09, 0.50) <0.01
Q4	0.37 (0.17, 0.58) <0.01	0.37 (0.16, 0.58) <0.01	0.33 (0.12, 0.54) <0.01
continuous	0.04 (0.02, 0.06) <0.01	0.04 (0.02, 0.05) <0.01	0.04 (0.02, 0.05) <0.01
P for trend	0.0002	0.0003	0.0007
**CERAD: Score Trial 3 Recall**			
**CDAI**			
Q1	Ref	Ref	Ref
Q2	0.08 (−0.12, 0.29) 0.42	0.09 (−0.11, 0.30) 0.37	0.09 (−0.11, 0.29) 0.39
Q3	0.30 (0.10, 0.50) <0.01	0.31 (0.11, 0.52) <0.01	0.29 (0.09, 0.50) <0.01
Q4	0.40 (0.20, 0.61) <0.01	0.41 (0.20, 0.62) <0.01	0.38 (0.17, 0.59) <0.01
continuous	0.04 (0.03, 0.06) <0.01	0.04 (0.03, 0.06) <0.01	0.04 (0.02, 0.06) <0.01
P for trend	<0.0001	<0.0001	<0.0001
**CERAD: Score Delayed Recall**			
**CDAI**			
Q1	Ref	Ref	Ref
Q2	0.21 (−0.04, 0.47) 0.10	0.21 (−0.05, 0.47) 0.10	0.22 (−0.03, 0.47) 0.09
Q3	0.44 (0.19, 0.70) <0.01	0.43 (0.18, 0.69) <0.01	0.44 (0.19, 0.70) <0.01
Q4	0.53 (0.27, 0.78) <0.01	0.52 (0.26, 0.78) <0.01	0.51 (0.25, 0.77) <0.01
continuous	0.04 (0.02, 0.06) <0.01	0.04 (0.02, 0.06) <0.01	0.04 (0.02, 0.06) <0.01
P for trend	<0.0001	<0.0001	<0.0001
**Animal Fluency: Score Total**			
**CDAI**			
Q1	Ref	Ref	Ref
Q2	0.98 (0.37, 1.59) <0.01	1.04 (0.42, 1.65) <0.01	1.09 (0.48, 1.71) <0.01
Q3	1.52 (0.92, 2.12) <0.01	1.62 (1.01, 2.23) <0.01	1.70 (1.09, 2.31) <0.01
Q4	2.04 (1.43, 2.65) <0.01	2.15 (1.53, 2.77) <0.01	2.19 (1.56, 2.81) <0.01
continuous	0.18 (0.13, 0.23) <0.01	0.19 (0.14, 0.24) <0.01	0.19 (0.13, 0.24) <0.01
P for trend	<0.0001	<0.0001	<0.0001
**Digit Symbol: Score**			
**CDAI**			
Q1	Ref	Ref	Ref
Q2	3.06 (1.13, 4.99) <0.01	3.12 (1.18, 5.05) <0.01	3.01 (1.12, 4.90) <0.01
Q3	6.19 (4.29, 8.09) <0.01	6.21 (4.29, 8.14) <0.01	5.91 (4.02, 7.81) <0.01
Q4	8.42 (6.49, 10.34) <0.01	8.39 (6.43, 10.35) <0.01	7.82 (5.89, 9.75) <0.01
continuous	0.62 (0.46, 0.78) <0.01	0.61 (0.45, 0.77) <0.01	0.55 (0.38, 0.71) <0.01
P for trend	<0.0001	<0.0001	<0.0001

Model 1 was not adjusted. Model 2 was adjusted for age, gender and race. Model 3 was adjusted for age, gender, race, education, marital status, Ratio of family income to poverty, Anti-hypertension therapy, Lipid-lowering therapy, Physical activity levels, body mass index, smoking status, total cholesterol, and glycohemoglobin.

Using two-piecewise linear regression models (Table 3). We identified the breakpoints in the link between CDAI and several cognitive function tests. The breakpoints were as follows: CERAD Trial 1 Recall: 1.23, Trial 2 Recall: 0.83, Trial 3 Recall: 4.1, Delayed Recall: 4.26, Animal Fluency: 5.8, and Digit Symbol: 5.08. The analysis indicated a significant positive impact of CDAI on cognitive test scores, with varying effects at different CDAI thresholds. Within lower CDAI ranges, the improvements in test scores were more noticeable, while the increases plateaued or even declined beyond the threshold. This suggests the potential role of dietary antioxidants in enhancing cognitive function, particularly within specific ranges.

**Table 3:** Threshold effect analysis of CDAI on cognitive tests using the two-piecewise linear regression model.

CDAI	CERAD: Score Trial 1 Recall	CERAD: Score Trial 2 Recall	CERAD: Score Trial 3 Recall	CERAD: Score Delayed Recall	Animal Fluency: Score Total	Digit Symbol: Score
Model I						
A Single Linear Effect	0.04 (0.03, 0.06) <0.0001	0.03 (0.02, 0.05) 0.0001	0.04 (0.02, 0.06) <0.0001	0.04 (0.01, 0.06) 0.0011	0.19 (0.14, 0.24) <0.0001	0.55 (0.39, 0.71) <0.0001
Model II						
Breakpoint (K)	1.23	0.83	4.1	4.26	5.8	5.08
For < K segment: Effect 1	0.08 (0.04, 0.11) <0.0001	0.08 (0.04, 0.12) 0.0002	0.06 (0.03, 0.09) <0.0001	0.09 (0.06, 0.12) <0.0001	0.28 (0.21, 0.35) <0.0001	1.04 (0.80, 1.27) <0.0001
For > K segment: Effect 2	0.02 (−0.00, 0.04) 0.0747	0.01 (−0.01, 0.04) 0.2580	0.02 (−0.01, 0.05) 0.2841	−0.03 (−0.07, 0.01) 0.1383	0.03 (−0.06, 0.13) 0.4940	−0.14 (−0.44, 0.15) 0.3405
Log-Likelihood Ratio Test	0.018	0.022	0.051	<0.001	<0.001	<0.001

Threshold effect analysis of CDAI on cognitive tests. Age, gender, race, education, marital status, Ratio of family income to poverty, body mass index, smoking status, total cholesterol and glycohemoglobin were adjusted.

### 3.3 Subgroup analysis

**Figure 3:**
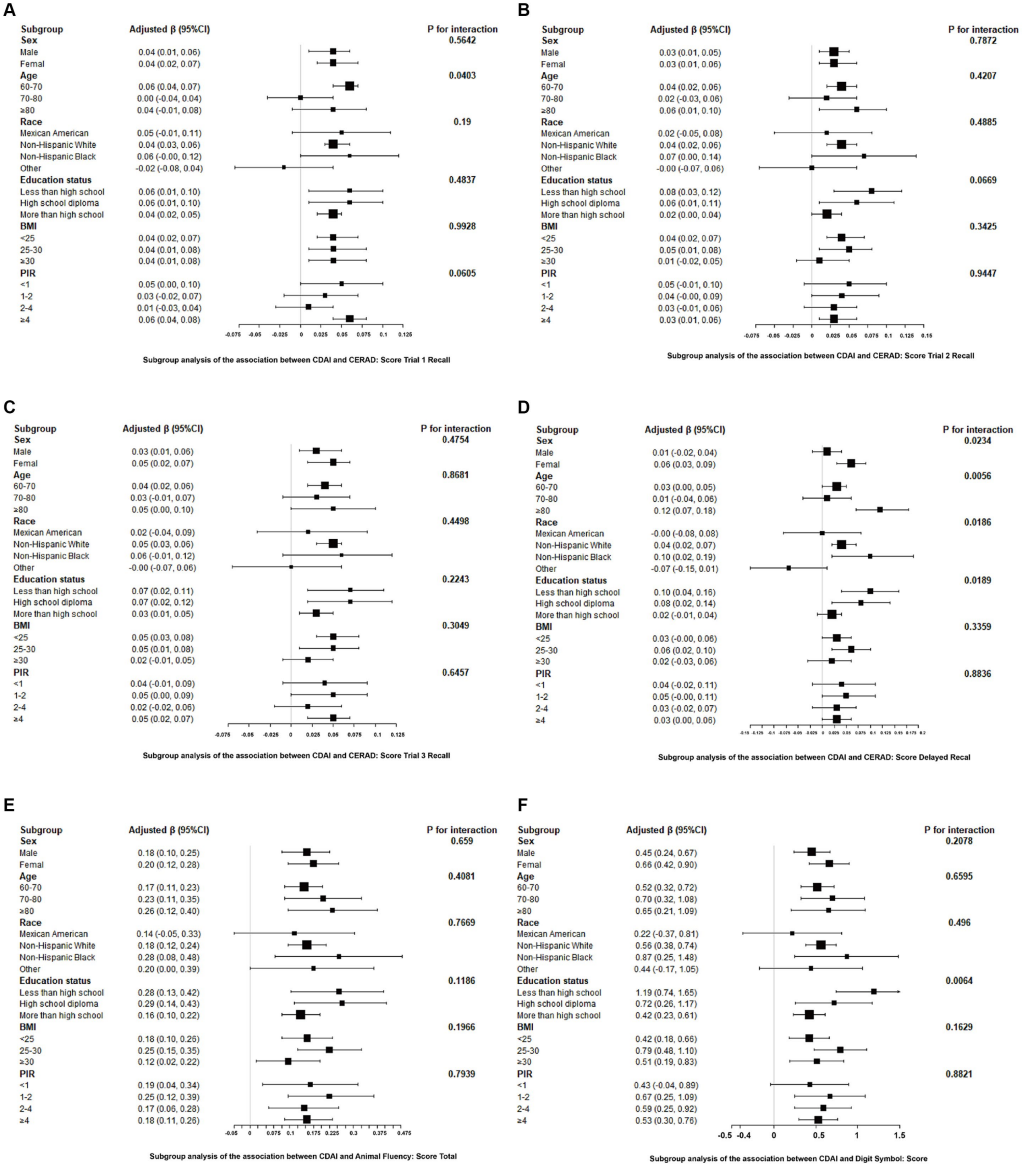
Associations between CDAI and cognitive tests stratified by age, sex, race, education, BMI, and PIR. Adjusted for age, gender, race, education, marital status, ratio of family income to poverty, body mass index, smoking status, total cholesterol, and glycohemoglobin. **(A)** CERAD: Score Trial 1 Recall, **(B)** CERAD: Score Trial 2 Recall, **(C)** CERAD: Score Trial 3 Recall, **(D)** CERAD: Score Delayed Recall, **(E)** Animal Fluency: Score Total, **(F)** Digit Symbol: Score.

After adjusting for covariates, the results from subgroup analyses, smoothing curve fitting, and generalized additive models indicated that CDAI had a universally positive impact on cognitive test scores, with more significant effects observed in women, those aged 80 and above, Non-Hispanic Black people, and individuals with lower education levels. This suggests that these groups may benefit more from higher dietary antioxidant intake. Differences among subgroups were mostly insignificant, indicating the consistency of CDAI benefits across diverse populations. Detailed information on subgroup analyses is provided in Figure 3.

## 4 Discussion

This cross-sectional study explored CDAI and cognitive function in older US adults using NHANES (2011–2014) data. It showed that higher CDAI correlated to better scores in memory, language, and executive function domains. Subgroup analyses further revealed more pronounced effects in women, those aged 80 and above, Non-Hispanic Black people, and individuals with lower education levels. However, given that subgroup analysis involves dividing the entire study sample into smaller subsets for analysis, this often leads to a decrease in statistical power, consequently impairing the ability to detect statistically significant outcomes. Accordingly, caution should be exercised when interpreting the results, and it is recommended that these findings be validated in future studies with larger sample sizes and pre-defined hypotheses to mitigate the risk of Type I errors.

Earlier research has demonstrated a beneficial relationship of total dietary antioxidant capacity (TAC) and cognitive function, even after adjusting for potential confounders, which aligns with our findings (21). Prospective cohort studies have shown that greater intake of antioxidant vitamins is linked to slower cognitive impairment and reduced likelihood of dementia (22, 23).

Vitamin A obtained from the diet accumulates in the liver as retinyl esters as well as releases gradually to ensure a steady supply of retinol to body cells, including those in the brain (24). The hippocampus, a critical area for cognition due to its role in learning and memory, requires vitamin A and retinoic acid to control the neuroplasticity necessary for these processes (25). Vitamin A is crucial for two aspects of neuroplasticity, long-term potentiation (LTP) and long-term depression (LTD), that are important to memory and recall. These reactions cause enduring alterations in synaptic strength, resulting in the enhancement or reduction of neuronal circuits. Synapses are the connections between neurons in the neural circuit, and changes in these circuits are thought to underlie learning and memory (24). In conclusion, these findings have provided significant insight toward the effect of vitamin A in supporting neuronal plasticity and cognitive function in adulthood.

Taking supplements of vitamin C and various antioxidants may play an essential role in maintaining cognitive abilities as we age. This effect is largely due to their capacity to combat oxidative stress, which is a major contributor to cellular aging, neurodegenerative disorders, and the decline in cognitive functions associated with aging (26, 27). Vitamin C is essential for the production and proper performance of both dopamine and norepinephrine in the brain (28). A study has demonstrated that high doses of vitamin C can significantly improve cognitive impairment in septic rats by reducing brain inflammation, protecting the blood–brain barrier, inhibiting oxidative stress, and activating the Nrf2/HO-1 signaling pathway (29). Additionally, vitamin C deficiency has been associated with hypoglycemia and cognitive impairment, primarily through S-nitrosylation-mediated activation of glycogen synthase kinase 3β, which regulates glucose homeostasis. This suggests that vitamin C supplementation may help prevent hypoglycemia and cognitive impairment in certain populations, particularly young women (18, 30). Research has also linked vitamin C deficiency to impairments in attention, executive function, recall, communication, and abstract thinking (31, 32). Overall, current evidence indicates that sustaining adequate vitamin C levels may aid in preventing cognitive loss due to age and neurological disorders, and vitamin C supplementation can enhance cognitive function.

Vitamin E, naturally present in the diet, has multiple bioactivities, including scavenging toxic free radicals as an antioxidant. As a potent lipid-soluble antioxidant, vitamin E is known for protecting against lipid peroxidation of the membranes of cells, which is essential to maintaining cognitive fitness (26, 33). Vitamin E can prevent lipid peroxidation by neutralizing lipid peroxyl radicals (LOO•), when vitamin E deficiency leads to increased lipid peroxidation in the nervous system, especially the oxidation of polyunsaturated fatty acids (such as DHA-PC), which are important components of nerve cell membranes. Increased lipid peroxidation can cause structural damage and dysfunction of nerve membranes, which in turn affect cognitive function. Studies have shown that vitamin E deficiency during embryonic development can lead to impaired neurodevelopment, lipid peroxidation and energy metabolism disorders, which affect the migration, proliferation, differentiation and survival of neural crest cells (34). These mechanisms underscore the critical role of vitamin E in neurological health. Moreover, it works in conjunction with other antioxidants, including selenium, vitamin C, and carotenoids, to safeguard cognitive health in older adults (35). Nevertheless, some systematic reviews have found that vitamin E does not enhance cognitive abilities among people via mild cognitive impairment (MCI) or dementia caused by Alzheimer’s disease (AD). Further study is needed to verify the inclusion of vitamin E supplements in dietary strategies designed to protect cognitive health in the elderly.

Selenium is a vital element necessary for sustaining mammalian life and is integrated into selenoproteins, that are crucial components within the body’s natural antioxidant system. The mind especially depends on a sufficient supply of selenium and is capable of preventing selenium deficiency (36, 37). Randomized controlled trials have demonstrated that administering high or super-nutritional doses of sodium selenate supplements can enhance selenium uptake in the central nervous system. In patients with AD, this has resulted in subtle yet major enhancements in the Mini-Mental Status Examination (MMSE), which evaluates aspects such as orientation in space and time, immediate and recall memory, calculation, comprehension, writing, and drawing to assess AD progression (38). Supplementing with selenium is a good option for alleviating certain symptoms of AD and MCI. Additional investigations will be needed on the long-term impacts of selenium supplementation.

Zinc is essential for growth, development, and healthy functioning within the immune system. Cognitive deficits and memory loss might occur as a result of zinc imperfections (39, 40). The potential role of zinc in dementia was first proposed by Burnet, Numerous original studies and meta-analyses have documented zinc’s role in AD pathology and its influence on cognitive function (41–43). ZnT and ZIP transporters precisely regulate zinc transport across neuronal membranes, thereby maintaining zinc homeostasis and regulating intracellular zinc concentrations. Once the function of these transporters is disrupted, zinc levels in the brain will fluctuate, which in turn affects the normal functioning of cell functions. ZIP7, in particular, locates in the endoplasmic reticulum - Golgi apparatus and is strongly associated with the regulation of metal homeostasis in neurodegenerative diseases such as Barton’s disease. Decreased expression of ZIP7 disrupts the balance of metals in cells, exacerbating cognitive decline in these diseases (44). A randomized, double-blind, and placebo-controlled investigation found that zinc supplementation in healthy adults aged 55–80 led to improved performance on two cognitive tests, specifically those assessing attention and spatial working memory (45). Another cross-sectional study showed both selenium and zinc intake were non-linearly related to cognitive function across all genders and that zinc and selenium consumption interacted to improve cognitive function, particularly in women (46). Additional research into the connections between zinc metabolism and neurological disorders could deepen our comprehension of the pathogenesis of such illnesses.

Carotenoids are natural pigments present in an array of vegetables and fruits, as well as in algae, plants, and photosynthesis-producing bacteria. Human beings are unable to generate carotenoids and have to get them through dietary sources or supplements (47). Carotenoids have shown potential effects on cognitive function, although their specific mechanisms of action are not well understood, they are presumed to be related to their antioxidant activity (48, 49). Carotenoids, such as lutein, zeaxanthin, and beta-carotene, significantly enhance cognitive function through a complex set of cellular and molecular mechanisms. At the core of these mechanisms are their potent antioxidant and anti-inflammatory properties, which effectively neutralize reactive oxygen species (ROS) and significantly reduce the level of oxidative stress within neurons, thereby protecting nerve cells from damage (50). Among them, lutein and zeaxanthin can also enhance neuroprotection by regulating gene expression closely related to oxidative stress response and cell survival. This process involves activation of the Nrf2 pathway, which is a key cellular defense against oxidative challenges and can up-regulate the expression of antioxidant enzymes and detoxification enzymes, thereby promoting the health and function of neurons (51). In addition, carotenoids support the maintenance of synaptic function by stabilizing neuronal membranes and promoting the expression of synaptic proteins such as brain-derived neurotrophic factor (BDNF). BDNF is essential for synaptic growth and long-term enhancement (LTP). Together, these physiological changes act on neural networks, laying a solid foundation for memory formation, consolidation, and overall cognitive performance improvement (52). A double-blind, controlled study revealed that prolonged intake of *β*-carotene (50 milligrams each day) played a role in sustaining cognitive function within a healthy general population. Participants showed significant positive changes in verbal memory, cognitive status, telephone interviews, and overall scores after an average of 18 years of treatment (53). Lutein and zeaxanthin, carotenoids with anti-inflammatory and antioxidant effects, have been connected with cognitive functions related to recall, processing quickness, focus, and logical thinking (49). Carotenoids are promising bioactive substances in the food chain that require further research to elucidate their health benefits, and adequate and optimal intake is recommended through food or supplements.

Since uncovering the link between free radicals and aging-related cellular and tissue damage, further research has highlighted oxidative harm as an important player in the onset of cardiovascular conditions, neurodegenerative diseases, and various cancers. Consequently, more people, particularly in developed nations, apply antioxidant supplements for better health and longevity (27). In 2004, Margaret E. Wright et al. introduced the concept of the CDAI, which considers the synergistic interactions between different molecules present in foods and summarizes a total of six dietary antioxidants: vitamins A, C, E, selenium, zinc, and carotenoids (15). Overall, a higher CDAI can be seen to be an indicator of a better lifestyle in general (with a high in vegetables and fruits).

The strengths of the research involve the use of the CDAI as a method for assessing the total antioxidant capacity of a diet. Furthermore, the analysis encompasses a wide and representative sample, accounts for numerous potential confounders, and incorporates various cognitive assessments related to neurodegenerative diseases like AD, including memory and executive function tests. Additionally, we used data extracted from the NHANES database were utilized, and survey-weighted methods were applied to achieve unbiased estimates.

However, this study also has several limitations. A major limitation is its cross-sectional design, which restricts the ability of the study to establish a causal relationship between CDAI and cognitive function. Given that longitudinal studies can enhance the robustness of causal inference by clarifying temporal sequences, reducing the possibility of reverse causality, and controlling for confounding factors, further research is needed to gain a deeper understanding of the causal relationship between CDAI and cognitive function. Additionally, the adoption of 24-h dietary recall may not accurately represent typical dietary patterns, thereby impacting the accuracy of the calculated dietary antioxidant levels. Due to the reliance on 24-h dietary recall data in this study, potential biases arise, such as underreporting or misreporting of dietary intake, which may have led to the underestimation or overestimation of the intake of certain nutrients or food categories in the results. To achieve more accurate and reliable dietary data, future research should explore the combined use of multiple methods, including repeated dietary recalls, biomarkers, and smart device assistance, in order to overcome the limitations inherent in a single approach. Furthermore, although this study has adjusted for several confounding factors, such as age, gender, and race, residual confounding may still exist due to unmeasured variables. Lastly, the NHANES dietary interview system was specifically designed for the U.S. population, and variations in growing environments might affect antioxidant levels, which could restrict the applicability of the results for different groups.

## 5 Conclusion

Its results indicate a significant positive correlation between the Composite Dietary Antioxidant Index (CDAI) and cognitive function in older individuals. Even after accounting for various confounders, higher CDAI scores were associated with better cognitive test performance. An antioxidant-rich diet may help safeguard cognitive health in the elderly.

## Data Availability

The original contributions presented in the study are included in the article/supplementary material, further inquiries can be directed to the corresponding author.

## Footnotes

^1^ https://www.cdc.gov/nchs/nhanes/

^2^ http://www.empowerstats.com

^3^ http://www.r-project.org
